# Growth and development of trabecular structure in the calcaneus of Japanese macaques (*Macaca fuscata*) reflects locomotor behavior, life history, and neuromuscular development

**DOI:** 10.1111/joa.13641

**Published:** 2022-02-17

**Authors:** Jaap P. P. Saers, Adam D. Gordon, Timothy M. Ryan, Jay T. Stock

**Affiliations:** ^1^ Department of Archaeology Cambridge University Cambridge UK; ^2^ Department of Anthropology University at Albany, SUNY Albany New York USA; ^3^ Department of Anthropology Pennsylvania State University State College Pennsylvania USA; ^4^ Department of Anthropology Western University London Ontario Canada

**Keywords:** calcaneus, life history, macaques, ontogeny, plasticity, trabecular bone

## Abstract

Bone structure dynamically adapts to its mechanical environment throughout ontogeny by altering the structure of trabecular bone, the three‐dimensional mesh‐like structure found underneath joint surfaces. Trabecular structure, then, can provide a record of variation in loading directions and magnitude; and in ontogenetic samples, it can potentially be used to track developmental shifts in limb posture. We aim to broaden the analysis of trabecular bone ontogeny by incorporating interactions between ontogenetic variation in locomotor repertoire, neuromuscular maturation, and life history. We examine the associations between these variables and age‐related variation in trabecular structure in the calcaneus of Japanese macaques (*Macaca fuscata*). We used high‐resolution micro‐computed tomography scanning to image the calcaneus in a cross‐sectional sample of 34 juvenile *M. fuscata* aged between 0 and 7 years old at the Primate Research Institute, Japan. We calculated whole bone averages of standard trabecular properties and generated whole‐bone morphometric maps of bone volume fraction and Young’s modulus. Trabecular structure becomes increasingly heterogeneous in older individuals. Bone volume fraction (BV/total volume [TV]) decreases during the first month of life and increases afterward, coinciding with the onset of independent locomotion in *M. fuscata*. At birth, primary Young’s modulus is oriented orthogonal to the ossification center, but after locomotor onset bone structure becomes stiffest in the direction of joint surfaces and muscle attachments. Age‐related variation in bone volume fraction is best predicted by an interaction between the estimated percentage of adult brain size, body mass, and locomotor onset. To explain our findings, we propose a model where interactions between age‐related increases in body weight and maturation of the neuromuscular system alter the loading environment of the calcaneus, to which the internal trabecular structure dynamically adapts. This model cannot be directly tested based on our cross‐sectional data. However, confirmation of the model by longitudinal experiments and in multiple species would show that trabecular structure can be used both to infer behavior from fossil morphology and serve as a valuable proxy for neuromuscular maturation and life history events like locomotor onset and the achievement of an adult‐like gait. This approach could significantly expand our knowledge of the biology and behavior of fossil species.

## Graphical abstract

Age‐related variation in trabecular bone structure of Japanese macaques is shaped by locomotor development, which, in turn, is shaped by interactions between neural circuits that develop in parallel to body size. These findings can be used to reconstruct locomotor ontogeny, neuromuscular maturation, and aspects of life history in fossil mammals.

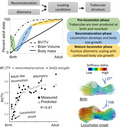

## 1 INTRODUCTION

Bone morphology is partially determined by genetic processes regulating growth and development, and partially by bone cells sensing and responding to their mechanical environment (Carter & Beaupré, 2001; Currey, 2002; Huiskes et al., 2000). The structure of spongy (trabecular) bone found inside bones is thought to be particularly responsive to mechanical stimuli (Carter et al., 1996; Carter & Beaupré, 2001; Kivell, 2016). The link between mechanical loading and the three‐dimensional (3D) structure of trabecular bone allows locomotor and postural behavior to be reconstructed in fossil taxa (Bardo et al., 2020; Barak et al., 2013; Bishop et al., 2018; Dunmore et al., 2020; Kivell, 2016; Ryan et al., 2018; Ryan & Ketcham, 2002; Skinner et al., 2015; Stephens et al., 2016; Zeininger et al., 2016). To understand how variation in trabecular structure arises within and between species, it is imperative to understand how it forms during growth and development (Gosman & Ketcham, 2009; Ryan et al., 2017; Ryan & Krovitz, 2006; Saers et al., 2020). Indeed, alterations to ontogenetic trajectories are the principal ways in which evolutionary changes in life history and morphology occur (Gould, 1977; Hallgŕimsson & Hall, 2005; Kardong, 2018; Woronowicz & Schneider, 2019). Recent methodological and technological advances in the analysis of trabecular bone structure have opened new possibilities for studying the development of trabecular bone structure (DeMars et al., 2020; Gross et al., 2014). Here we apply these new techniques to analyze the ontogeny of trabecular structure in the calcaneus of Japanese macaques (*Macaca fuscata*).

The musculoskeletal system undergoes striking changes throughout growth and development. Movements starting in utero and continuously changing throughout development, generate loads that shape bone morphology into the general adult form (Carter & Beaupré, 2001). These processes are essential for generating the adult morphology that is required for typical species‐specific gait and posture (Tardieu, 1999). Mammalian locomotion usually develops in a stereotypical species‐specific sequence of events that dramatically change how their skeletons are loaded (Doran, 1997; Lacquaniti et al., 2012; Sarringhaus et al., 2014). Changes in loading direction, magnitude, and frequency (Barak et al., 2011; Rubin et al., 2002; Sugiyama et al., 2010) alter the trabecular structure during development so that the structure provides a “functional record” of behavioral changes throughout development (Barak et al., 2011; Gosman & Ketcham, 2009; Pontzer et al., 2006; Ryan et al., 2017; Ryan & Krovitz, 2006; Saers et al., 2020, 2021; Tsegai et al., 2018; Wolschrijn & Weijs, 2004). Although the study of trabecular bone development is in its infancy, previous research on humans (Acquaah et al., 2015; Colombo et al., 2019; Cunningham & Black, 2009; Gosman & Ketcham, 2009; Raichlen et al., 2015; Reissis & Abel, 2012; Ryan et al., 2017; Ryan & Krovitz, 2006; Saers et al., 2020), great apes (Ragni, 2020; Tsegai et al., 2018), and other mammals (Gorissen et al., 2016, 2018; Tanck et al., 2001; Wolschrijn & Weijs, 2004) suggests a strong link between changes in loading conditions as gait develops and responses in trabecular structure.

### 1.1 Trabecular bone ontogeny

The connections between trabecular morphology and habitual loading patterns have been demonstrated experimentally (Barak et al., 2011; Biewener et al., 1996), but trabecular bone ontogeny is still not yet thoroughly understood, particularly how the degree of plasticity versus genetic canalization varies throughout development and into adulthood (Cunningham & Black, 2009; Gorissen et al., 2016; Raichlen et al., 2015; Reissis & Abel, 2012; Ryan et al., 2017; Saers et al., 2020). Bone growth occurs via the transformation of growth plate cartilage into bone through a series of cell and matrix changes (Burr & Organ, 2017; Byers et al., 2000; Parfitt et al., 2000). The transformation from growth plate cartilage to trabecular bone is similar among mammals, indicating a highly conserved process (Byers et al., 2000; Frost & Jee, 1994). This process sets up a basic trabecular structure which can later be modified through metabolic and mechanical factors. Trabecular bone is laid out orthogonal to the growth plate in a dense and anisotropic structure which is later refined into bone‐ and species‐specific heterogeneous adult states. Frost and Jee (1994) argue that the effects of mechanical usage during periods of rapid bone growth in early ontogeny explain many of the features observed during the ossification process. They propose that mechanical strain is the controlling mechanism for endochondral ossification, in which the underloaded elements of the dense bone structure during the first years of life are removed and bone is added in strained areas, resulting in a mechanically adapted state (Frost & Jee, 1994). This model correctly predicts observations of bone loss at early stages of ontogeny and explains it as the result of the removal of redundant material below a certain strain threshold (Carter et al. 1991, 1996; Carter & Beaupré, 2001; Frost, 2003; Pivonka et al., 2018).

### 1.2 The brain–bone connection

Brains and trabecular bone have more in common than one might initially think. Both are made up of complex, interconnected 3D structures and broadly share developmental patterns. At birth, both trabeculae and neurons are overproduced (Collin & Van Den Heuvel, 2013; Rabinowicz et al., 1996). Structures are refined to a more heterogeneous state during ontogeny through modeling in bone and synaptic pruning in neurons (Sakai, 2020). This happens under the influence of some input, presumably mechanical in terms of trabecular bone (Carter & Beaupré, 2001; Huiskes et al., 2000), and through neural activity in the brain (Sakai, 2020; Shatz, 1990). In both cases, there is a long history of debate as to how much of its respective morphology is genetically canalized versus plastic in response to its environment, that is, nature versus nurture, and in both cases, the consensus is “both.” While starting with an excess of connections to remove many of them later may seem inefficient, the result is a state that is adapted to an individual’s specific environment. Indeed, this process is so efficient that it is found in many other tissues as well including connective tissues like ligaments and tendons (Grinnell, 2000) to the nervous system (Sakai, 2020).

The patterns of the growth and development of trabecular structure reviewed above are consistent with a model where a generalized trabecular structure is formed by dynamic adaptation to local, bone‐ and region‐specific loading patterns. These loading patterns are generated by neural circuits that develop in parallel to increases in physical size and weight of a growing organism (Forssberg, 1985). Locomotor patterns are transformed from an immature state to increasingly adult‐like patterns during development. During the early ontogeny of gait, infants are mainly focused on minimizing the risk of falling. When individuals increase in strength, stability improves, and postural constraints are reduced (Vaughan & Langerak, 2003). It is thought that development subsequently proceeds to select the most optimal neural networks (Forssberg, 1999), resulting in a reduction in the variability of muscle activation and co‐contraction, and the adult gait pattern emerges (Okamoto et al., 2003). If trabecular structure is a reliable reflection of gait mechanics, then changes in trabecular structure during growth should reflect gait mechanics, which in turn reflects the degree of neurological maturation of locomotion, as well as an animal’s degree of precociality. If this link can be demonstrated, then trabecular structure could be a valuable proxy for neuromuscular maturation in fossil species.

Across mammals (Garwicz et al., 2009) and birds (Iwaniuk & Nelson, 2003), adult brain size strongly predicts time to locomotor onset after conception. In addition, the onset of walking is strongly correlated with the timing of several important aspects of brain development. In humans, locomotion is not just a developmental precursor to numerous psychological changes but plays a causal role in their formation (Anderson et al., 2013; Campos et al., 2000; Dahl et al., 2013; Uchiyama et al., 2008). The onset of human independent locomotion is followed by a revolution in perception‐action coupling, spatial cognition, memory, and social and emotional development (Anderson et al., 2013). Research indicates that neural function and structure reciprocally influence one another throughout development (Anderson et al., 2013; Campos et al., 2000), placing the activity of locomotor development in the center of development, rather than being just a consequence of neural maturation. In other words, the onset of independent locomotion is an important life history event related to adult brain size and the timing of neuromuscular development. If we can detect bony markers of locomotor development, this would be able to provide a unique insight into fossil locomotion as well as aspects of life history (Zihlman, 1992).

### 1.3 Locomotor development in Japanese macaques

The basic locomotor characteristics of Japanese macaques appear in the first 2 months after birth (Dunbar & Badam, 1998; Kimura, 2000; Nakano, 1996; Torigoe, 1984). Newborns cannot walk, stand, or sit on their own and reflexively hang on to their mother for transport. Initial, somewhat poorly coordinated quadrupedal movements emerge in the second half of the first month (Nakano, 1996; Torigoe, 1984). Macaques begin to locomote primarily by walking in both diagonal and lateral sequences, followed after 4 weeks by occasionally running and trotting (Nakano, 1996). Independent locomotion away from the mother becomes regular after 2 months of age. Coordinated walking appears after 3 months. Prior to this, their style of walking is limited by the immature development of their musculoskeletal system (Nakano, 1996). Locomotion becomes increasingly refined and independent throughout the first year of life (Dunbar & Badam, 1998). Locomotor activities include unskilled locomotion between 1 and 6 months when monkeys still frequently lose their footing. After 6 months, the macaques are skilled at both terrestrial and arboreal locomotion (Kimura, 2000; Nakano, 1996; Torigoe,, 1984).

Between the age of 1‐ and 3‐years macaques enter the juvenile phase which contains the most diverse range of posture and locomotion. Juveniles have a well‐developed musculoskeletal system which, combined with a small body size, enables juvenile macaques to exploit terrestrial and arboreal environments to their fullest potential (Dunbar & Badam 1998). After the juvenile phase macaques are considered adults but they continue to grow in size, albeit at a decreasing rate, until around 10 years of age (Hamada et al., 2004). The postural and locomotor repertoires of adults are reduced compared to juveniles, potentially due to increases in body size. The largest reduction is among play behaviors in the small‐branch setting of trees, and below‐branch postures and locomotion disappear (Dunbar & Badam, 1998). Passive joint mobility of macaques declines rapidly between 6 and 15 months and more gradually afterward (DeRousseau et al., 1983;Turnquist & Wells, 1993; Wells & Turnquist, 2001). These studies on the ontogeny of joint mobility, postural, and locomotor behaviors indicate that the most substantial changes in locomotor anatomy and behavioral control occur within the first 18 months of life (Turnquist & Wells, 1993; Wells & Turnquist, 2001).

### Aims

If trabecular bone markers of behavior, neuromaturation, and life history variables such as the onset of independent locomotion can be established, reconstructions of the biology and behavior of fossil animals could be substantially improved. Our aim is to first document how trabecular structure of the calcaneus of Japanese macaques varies with age and body mass. We then test whether landmark events in the development of locomotion (independent locomotion, achievement of adult‐like locomotor repertoires) in macaques coincide with clear signals in the trabecular structure. We do this by analyzing whole‐bone averages of standard trabecular properties (Table 1) as well as regional variation in the distribution of these properties throughout the calcaneus. Additionally, we aim to broaden the analysis of bone structure beyond pure locomotor mechanics by proposing a new way to incorporate the interactions between behavior and neuromuscular development, body size, and life history.

**TABLE 1:** Definitions of trabecular bone properties

Measurement	Abv.	Description
Bone volume fraction	BV/TV	Ratio of bone volume to total volume of interest
Trabecular thickness	Tb.Th (mm)	Average trabecular strut thickness
Trabecular separation	Tb.Sp (mm)	Average distance between struts
Primary Young’s modulus	*E* (MPa)	The tensile stiffness of a solid material in the direction in which it is stiffest measured in a sphere

### 1.4 Predictions

If the development of trabecular structure is largely or partially mediated through mechanics (Carter & Beaupré, 2001; Huiskes et al., 2000) rather than genetic programming (Lovejoy et al., 2003), one would predict that the minimal locomotor‐related loading during the first month might lead to either bone resorption or no change in bone volume (BV) relative to total volume (TV), while increases in loading after the onset of locomotion should result in bone formation (Frost, 2003; Pivonka et al., 2018). New mechanical stimulation after the onset of locomotion combined with increases in body size are predicted to initiate reorganization of the trabecular architecture throughout the calcaneus. Redundant trabeculae are expected to be removed, while trabeculae oriented in directions involved in the distribution of loads associated with locomotion are preserved or enlarged, resulting in a reorganization of the primary direction of bone stiffness, and increases in bone volume fraction and average trabecular thickness (Tb.Th). After the onset of locomotion, trabeculae are expected to increase in regional variation in the amount of bone, bone stiffness, and average orientation of trabeculae. The highest bone volume fraction (BV/TV) is expected to be found where loads are applied to the calcaneus, including under joint surfaces (posterior talar facet and calcaneocuboid joint) and the attachment sites of the Achilles tendon and the plantar ligaments (Giddings et al., 2000; Saers et al., 2020).

We predict the following events to invoke the following associated morphological signals:

1. Onset of locomotion: Whole‐bone average BV/TV is expected to decrease before the onset of locomotion and increase afterward. As such, the slope of the relationship between BV/TV and age will shift from negative to positive, trabeculae will become thicker, and primary stiffness will align within the direction of joint loading (Barak et al., 2011; Ryan et al., 2017; Saers et al., 2020).
2. Appearance of adult‐like locomotor repertoire: After locomotion has matured the only remaining effects on trabecular structure should be allometry as body size continues to increase. The slope of the relationship between BV/TV and age should become more shallow and follow allometric scaling with still increasing body size (Doube et al., 2011; Mulder et al., 2020; Ryan & Shaw, 2013; Saers et al., 2019). Based on our review of the ontogeny of joint mobility, postural, and locomotor behaviors above, we predict this change to a shallower slope after 18 months (Turnquist & Wells, 1993; Wells & Turnquist, 2001).
3. Neuromuscular maturation of gait: age‐related variation in loading patterns is generated by neural circuits that develop in parallel to increases in physical size and weight of a growing organism. As such, trabecular properties should be predicted by an interaction between body mass and neuromaturation. Here we use the estimates of the percentage of adult brain size for age as a proxy for neuromuscular maturation.

## 2 MATERIALS AND METHODS

### 2.1 Sample

We used high‐resolution micro computed tomography (μCT) scanning to image the calcaneus from the skeletal remains of 34 juvenile male Japanese macaques (*Macaca fuscata fuscata*) from a colony housed in a large open‐air enclosure at the Primate Research Institute, University of Kyoto (PRI) (Torigoe, 1984). The enclosure was designed to mimic the natural environment of Japanese macaques and contains plentiful trees and climbing installations. Individuals are of known age and body weight at death, but the cause of death was not recorded. Specimens were μCT scanned at the PRI with a SkyScan1275 μCT scanner at 95 kV and 95 μA for 2400 projections with an exposure of 0.216 s. CT scans were saved as 16 bit tiff stacks with isotropic voxel dimensions between 16 and 22 μm depending on bone size. We tested for potential effects of variation in voxel dimensions by artificially reducing the voxel size from 16 to 22 μm in Amira 6.7.0 (Thermo Fisher Scientific) using the resample module with a Lanczos filter. Least‐squares linear regression analysis was run for each variable to test for significant influences of voxel size on trabecular properties. Downsampling individuals from 16 to 22 μm yielded identical results, indicating the voxel size differences do not affect the results. We calculated percentage of adult body mass for age in *Macaca fuscata* based on data from Hamada (1994) and percentage brain volume for age by combining data from two studies (DeSilva & Lesnik, 2008; Van Minh & Hamada, 2017) and dividing by the average adult brain volume (105.6 cm^3^, DeSilva & Lesnik, 2008). When then fit the following curve to predict percentage of adult brain volume for age in months: 0.7417 × age^0.0681^.

### 2.2 Calculation of whole bone average trabecular properties

The 3D structure of trabecular bone was quantified using standard trabecular properties (Table 1) using ORS Dragonfly (Object Research Systems [ORS] Inc., www.theobjects.com/dragonfly). Tiff stacks were segmented into cortical and trabecular bone using the method described in Kohler et al. (2007), following recommendations by (Bouxsein et al., 2010). We calculated the average bone volume fraction (BV/TV), Tb.Th, and trabecular separation (Tb.Sp).

### 2.3 Three‐dimensional mapping of trabecular structure

**FIGURE 1:**
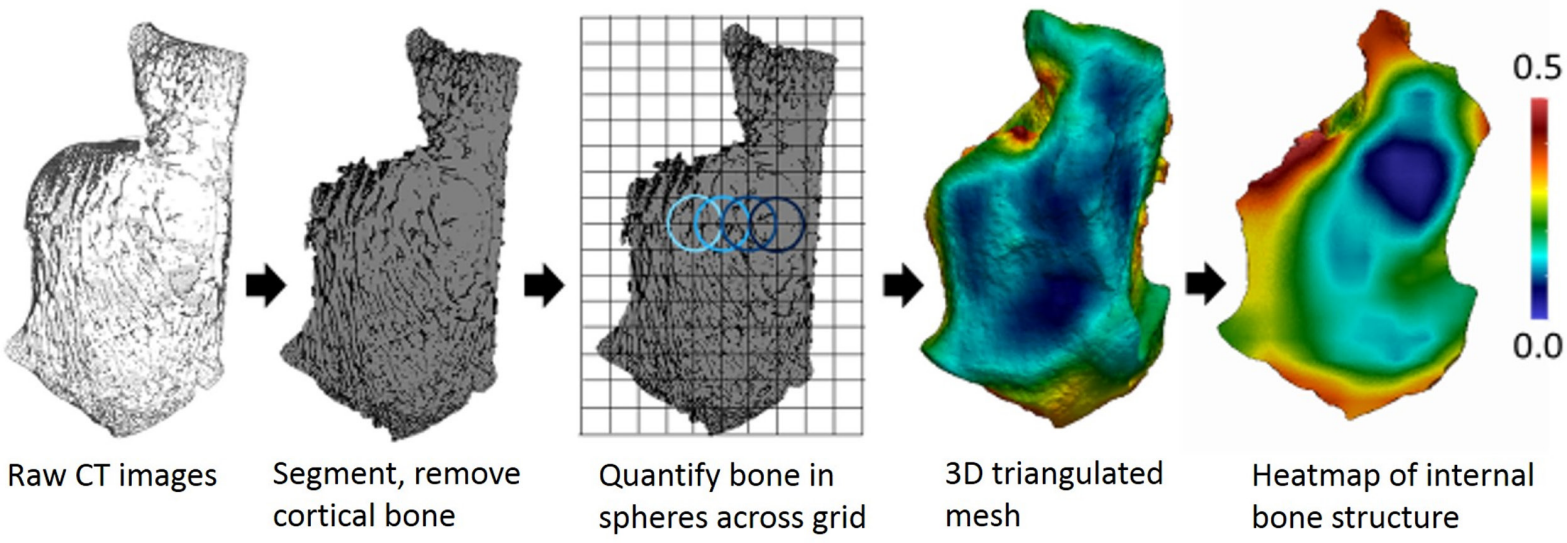
Medtool workflow example showing a sagittal slice through a calcaneus

Segmented scans were categorized into three regions (cortex, trabeculae, and internal region of the bone) using Medtool 4.0 (www.dr‐pahr.at, Figure 1). Morphometric maps of BV/TV and primary Young’s modulus were generated following Gross et al. (2014). A 3D tetrahedral mesh was created of the internal region of the bone using CGAL (http://www. cgal.org). A mesh size of 0.6 mm was used. A 3.5 mm background grid was applied in three dimensions to the trabecular, and BV/TV and Young’s modulus were quantified at each node of the background grid using a 7 mm sampling sphere. The values from each sampling sphere were interpolated and applied to elements of the 3D tetrahedral mesh to generate morphometric maps. Changes in the orientation of average primary Young’s Modulus were investigated using a spherical volume of interest with a diameter of 50% of the maximum posterior talar facet length (Figure 8).

### 2.4 Statistical analysis

Linear regressions and interactions between trabecular properties, age, and body weight were performed in R version 4.0.2 (R Core Team, 2021). Alpha level was set to 0.05 for all statistical tests. When comparing various regression models, the model with the lowest Akaike Information Criterion (AIC; Akaike, 1974) and highest *R* ^2^ was chosen as indicating the highest model quality. Adding additional variables to a regression always increases the fit (*R* ^2^) due to spurious correlations, this process is called overfitting and causes the model to learn too much from the data, resulting in poor predictive power for non‐measured samples. AIC measures the degree to which a model is overfit with lower values indicating a greater model quality (Akaike, 1974; McElreath, 2015). In addition to regular linear regression, we run piecewise regressions using the ‘segmented’ R package (Muggeo, 2008). Piecewise regression is a useful technique for finding significant changes in slope in the relation between a dependent and independent variable. The technique uses dummy variables and an interaction term to split a linear regression into multiple segments. The least‐squares method is applied separately to each segment, by which the two regression lines are made to fit the dataset as closely as possible while minimizing the sum of squares of the differences between observed and predicted values of the dependent variable. We compared models with 0, 1, or 2 segments and chose the model with the highest *R* ^2^ and lowest AIC as the highest quality model.

## 3 RESULTS

### 3.1 Bone properties with age

**FIGURE 2:**
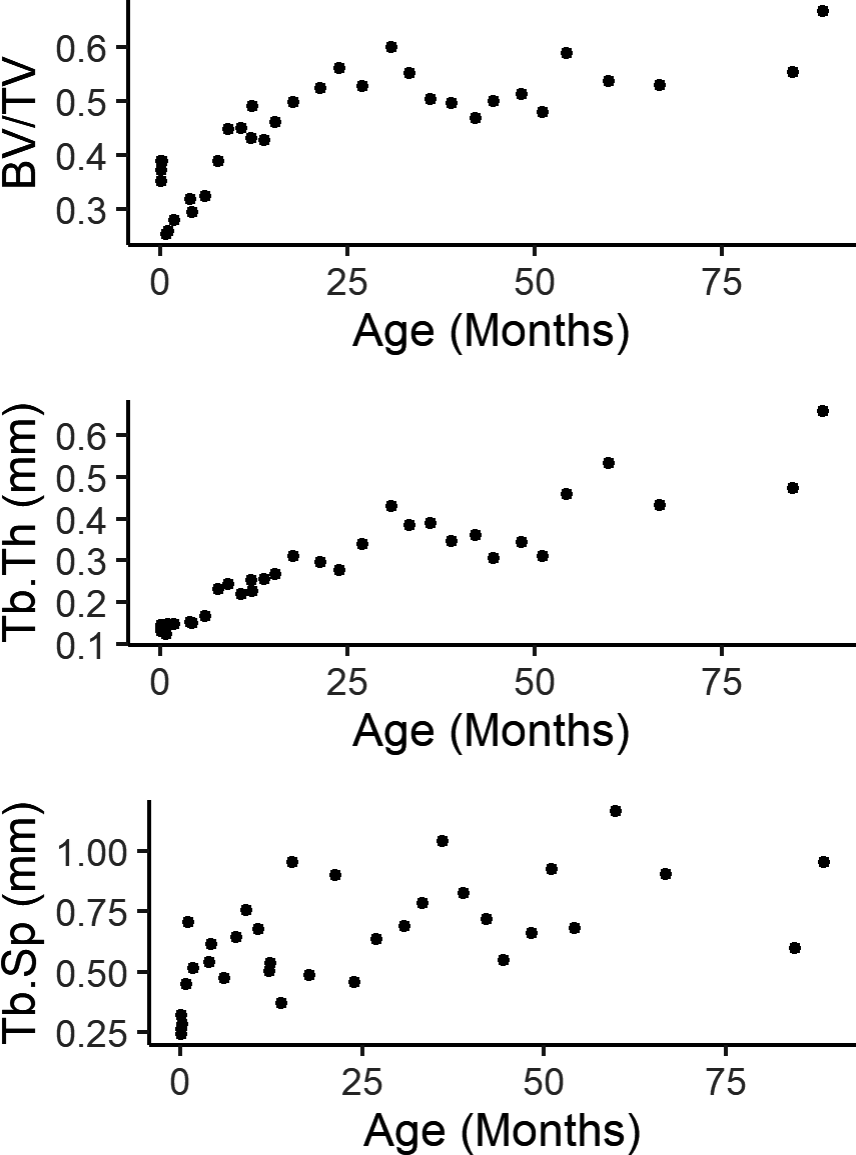
Whole bone trabecular properties plotted against age in months. *n* = 34. BV/TV, bone volume fraction; Tb.Th, trabecular thickness; Tb.Sp, trabecular separation

**FIGURE 3:**
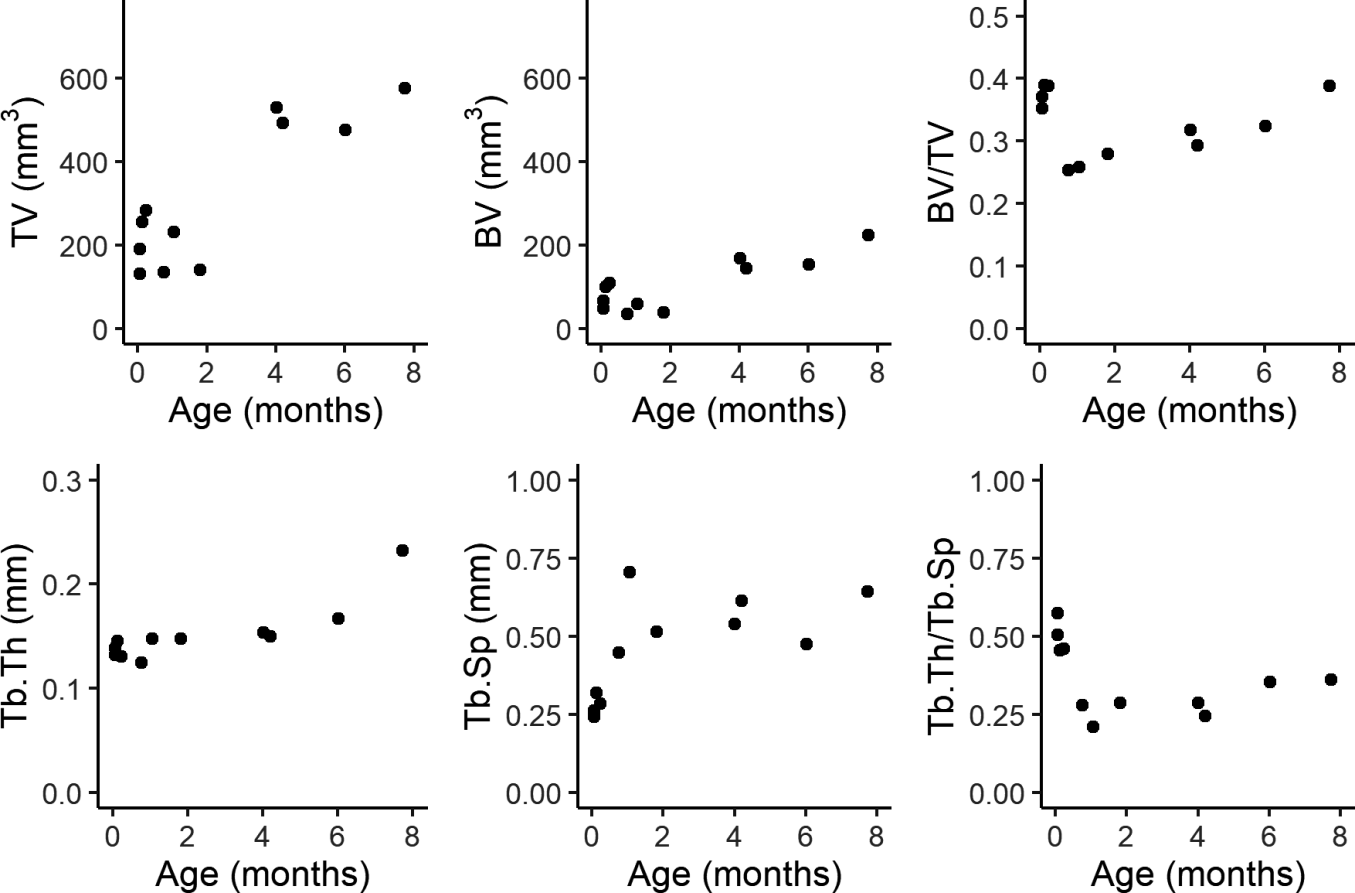
Whole bone trabecular properties plotted against age during the first 8 months of life. *n* = 34. BV/TV, bone volume fraction; Tb.Th, trabecular thickness; Tb.Sp, trabecular separation

Mean trabecular properties calculated in the whole calcaneus are plotted against age in Figure 2 and trends during the first 8 month of life in Figure 3. At birth, BV/TV is relatively high followed by a sharp decline in BV/TV during the first month. BV/TV gradually begins to increase between the first and second months for roughly 2 years after which BV/TV flattens out. Tb.Th and Tb.Sp increase gradually with age from birth to 7 years of age (the maximum age of juveniles in the sample). The drop in BV/TV is correlated with an increased average distance between trabeculae (Tb.Sp) while mean trabecular thickness remains constant. After the first month of life, when macaques start to locomote somewhat regularly, BV/TV rises in concert with Tb.Th and keeping a relatively constant Tb.Sp. Indeed, change in BV/TV is very strongly correlated to Tb.Th (linear regression *R* ^2^ = 0.77, *p* < 0.001, AIC = −102.5). Adding an interaction between Tb.Th and Tb.Sp increases *R* ^2^ to 0.81 and has a slightly lower AIC of −105.7, indicating a higher model quality. These data suggest that much of the variation in BV/TV can be explained by an interaction between average trabecular thickness and average distance between trabeculae. Figure 3 also shows that a reduction in BV/TV in the first month is driven by a reduction in bone volume (BV) and not by an increase in TV. The trend in Tb.Th/Tb.Sp shows a clear reduction in average Tb.Th relative to Tb.Sp. At birth the average Tb.Sp is roughly double that of the average Tb.Th. After 1 month, the average Tb.Sp is around four times higher than the average Tb.Th while Tb.Th remains unchanged.

**FIGURE 4:**
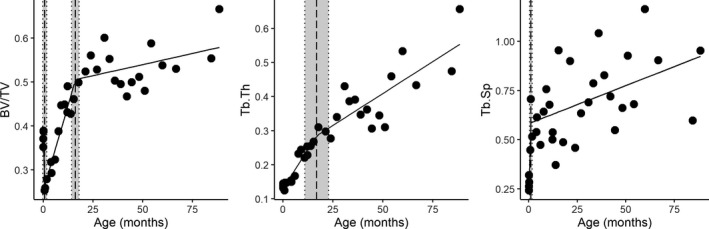
Trabecular properties against age in months. Piecewise regression line segments are plotted as solid lines. The vertical dashed lines represent the maximum likelihood estimate of the break points, and the dotted vertical lines represent the standard error of the estimate. *n* = 34. BV/TV, bone volume fraction; Tb.Th, trabecular thickness; Tb.Sp, trabecular separation

We performed piecewise regressions and used maximum likelihood to identify significant breaks in the relationship between age and trabecular properties (Figure 4, Table 2). For BV/TV, a piecewise regression model with two break points has a higher quality (adj. *R* ^2^ = 0.85, AIC = −115.0) compared to a regular linear regression model (*R* ^2^ = 0.61, AIC = −84.4). Break points were identified at 0.8 months with a standard error of 1.0, and one at 16.2 months with a standard error of 1.9. For Tb.Th, a model with a single break has the highest quality (adj. *R* ^2^ = 0.88, lowest AIC = −107.2), and fits better than the standard regression model (*R* ^2^ = 0.85, AIC = −102.1). Here the breakpoint is identified at 16.9 months with a standard error of 5.9 months. This break point overlaps with the second break point identified for BV/TV. For Tb.Sp, a piecewise regression model with one break point has is the highest quality model (*R* ^2^ = 0.48, AIC = −20.0) The breakpoint occurs at 1.1 months with a standard error of 0.7 months and overlaps with the first break point identified for BV/TV. Overall, all the analyses reported here find significant breaks in the relationships between trabecular bone properties and age around two age points. The first break point (0.8–1.1 months) coincides with the period of locomotor onset in Japanese macaques which occurs on average around the end of the first months of life. The second break point, around 16–17 months, corresponds to the end of the period of the greatest changes in locomotor anatomy and behavioral control (12–18 months of life, Turnquist & Wells, 1993, Wells & Turnquist, 2001) and the eruption of the first permanent molars (Smith et al., 1994).

**TABLE 2:** Piecewise regression model selection

Dependent variable	Number of breaks	Break points (age in months ± SE)	Adj. *R* ^2^	AIC	*p*
BV/TV	0		0.61	‐84.4	<0.001
	1	19.6 ± 3.9	0.77	−102.4	<0.001
	**2**	**0.8 ± 1.0**, **16.2 ± 1.9**	**0.85**	**−115.0**	**<0.001**
Tb.Th	0		0.85	−102.1	<0.001
	**1**	**16.8 ± 5.9**	**0.88**	**−107.2**	**<0.001**
	2	No additional breaks could be estimated			
Tb.Sp	0		0.36	−13.9	0.002
	**1**	**1.1 ± 0.7**	**0.48**	**−20.0**	0.031
	2	1.1 ± 0.8, 60.0 ± 26.0	0.48	−18.5	0.028

*Note*: Independent variable is age, dependent variable are trabecular properties, *n* = 34. 0 breaks = standard linear regression, 1 break = piecewise regression with one inflection point, 2 breaks = piecewise regression with two inflection points. Highest quality models in bold.

Abbreviations: AIC, Akaike Information Criterion; BV/TV, bone volume fraction; Tb.Th, trabecular thickness; Tb.Sp, trabecular separation; SE, standard error.

### 3.2 Bone properties with body weight

**FIGURE 5:**
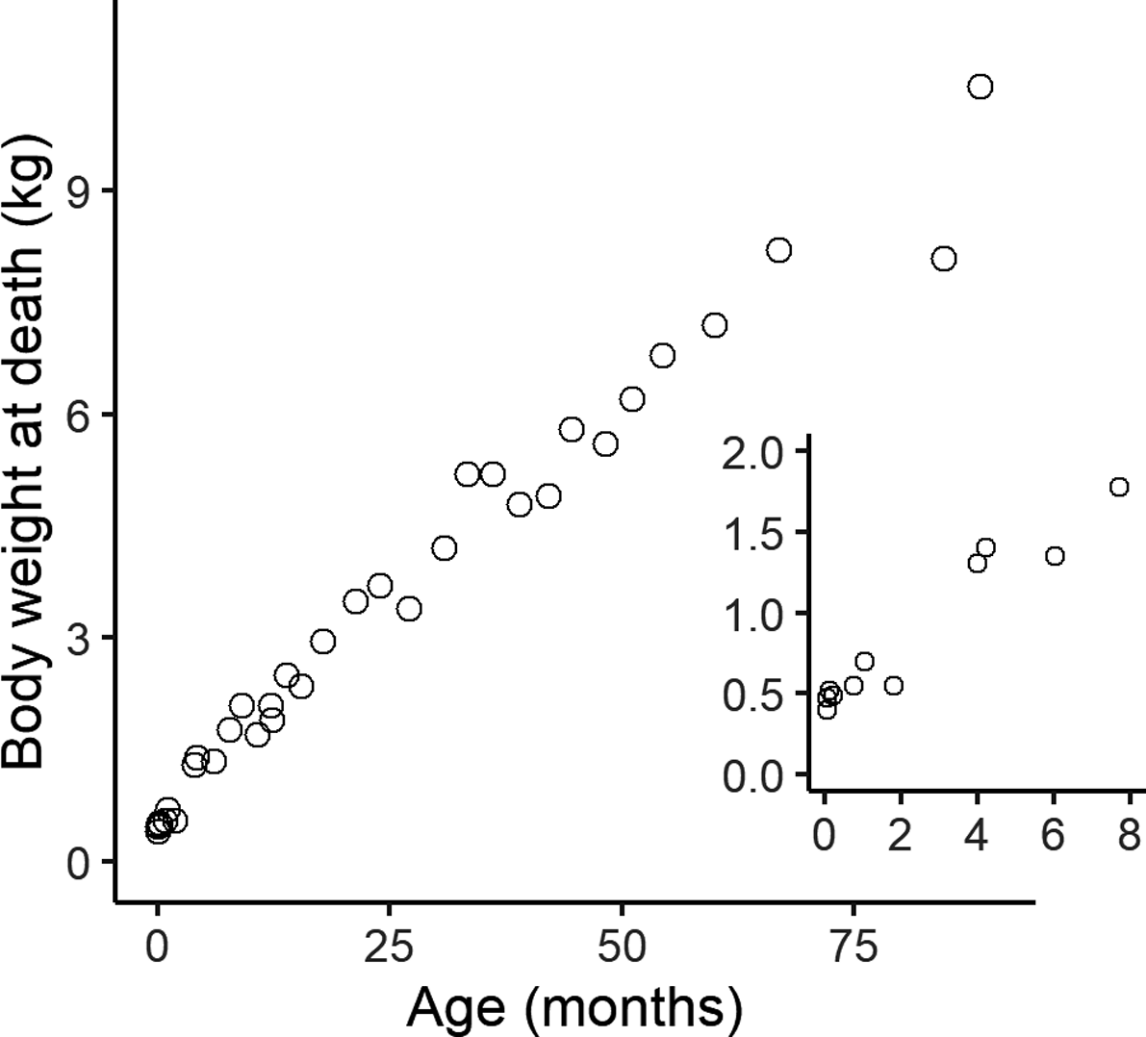
Body weight at death plotted against age with a closeup detailing the first 8months. *n* = 34

**FIGURE 6:**
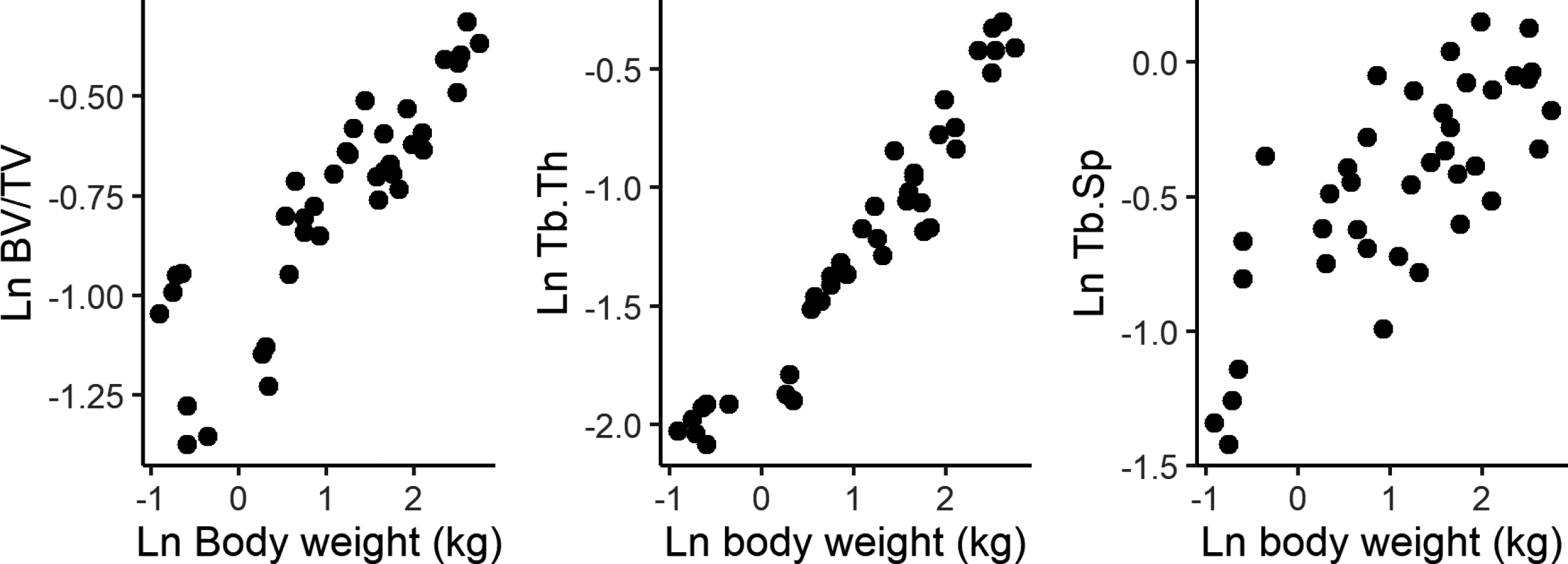
Natural log of body weight at death plotted against the natural log of trabecular properties. *n* = 34. BV/TV, bone volume fraction; Tb.Th, trabecular thickness; Tb.Sp, trabecular separation

The relationship between age and body weight at death is plotted in Figure 5. Log–log plots of trabecular properties against body weight at death are shown in Figure 6. Regression coefficients for a linear model with body weight as a predictor and trabecular properties as dependent variables are provided in Table 3. Significant positive relationships were found between body weight and BV/TV, Tb.Sp, and Tb.Th. For Tb.Th and Tb.Sp the predicted isometric scaling coefficient is 1/3 as body mass scales volumetrically (to the third power) and trabecular thickness scales by length (to the first power). As BV/TV is a ratio it should scale with a slope of 0 under isometry. BV/TV and Tb.Th scale with positive allometry (Table 2) while Tb.Sp scales isometrically.

**TABLE 3:** Regression coefficients of the natural logarithm (ln) of trabecular properties as dependent variable and ln body mass as the predictor, *n* = 34

Dependent variable	Intercept	Slope	Slope SE	df	*p*	*R* ^2^	AIC	Allometry
Ln BV/TV	−1.008	0.215	0.025	32	<0.001	0.69	−32.92	Positive
Ln Tb.Th	−1.746	0.457	0.025	32	<0.001	0.91	−33.71	Positive
Ln Tb.Sp	−0.777	0.300	0.049	32	<0.001	0.54	11.37	Isometric

Abbreviations: AIC, Akaike Information Criterion; BV/TV, bone volume/total volume; SE, standard error; Tb.Th, average trabecular thickness (mm); Tb.Sp, average distance between trabeculae (mm).

Regression parameters for models where trabecular properties are predicted by the interaction between body weight and locomotor onset are given in Table 4. Individuals younger than 1 month are partially dependent on their mothers for locomotion and therefore coded as pre‐locomotor onset, and those older than 1 month were coded as post‐locomotor onset (Torigoe, 1984). The linear models with an interaction between body mass and locomotor onset given in Table 4 have higher *R* ^2^ and lower AIC for BV/TV, Tb.Th, and Tb.Sp compared to the univariate linear models. This indicates that the interaction models have a greater fit to the data while at the same time not being overfit compared to the univariate models. BV/TV scales with a negative slope before locomotor onset and with a positive slope afterward. Tb.Sp scales with a positive slope before and after locomotor onset but the slope is significantly shallower afterward.

**TABLE 4:** Comparison of various regression models for each trabecular property, the best fit (highest *R* ^2^) and highest model quality (lowest AIC) are in bold

Dependent	Independent	Adj. *R* ^2^	AIC	*p*
BV/TV	BM	0.76	−103.6	<0.001
	BM * onset	0.75	−101.2	<0.001
	BM * percent adult brain size	0.81	−112.5	<0.001
	BM * percent adult brain size * onset	**0.87**	**−122.4**	**<0.001**
Tb.Th	BM	0.93	−119.7	<0.001
	BM * onset	0.92	−117.8	<0.001
	BM * percent adult brain size	**0.93**	**−120.4**	**<0.001**
	BM * percent adult brain size * onset	0.92	−114.3	<0.001
Tb.Sp	BM	0.42	−17.5	<0.001
	**BM × onset**	**0.53**	**−24.6**	**<0.001**
	BM * percent adult brain size	0.52	−24.4	<0.001
	BM * percent adult brain size * onset	0.48	−17.7	<0.001

*Note*: *N* = 34. Onset = older than 2 months, independent if older than 2 months.

Abbreviations: AIC, Akaike Information Criterion; BM, body mass at death (kg); BV/TV, bone volume/total volume; Tb.Th, average trabecular thickness (mm); Tb.Sp, average distance between trabeculae (mm).

### 3.3 Morphometric maps

**FIGURE 7:**
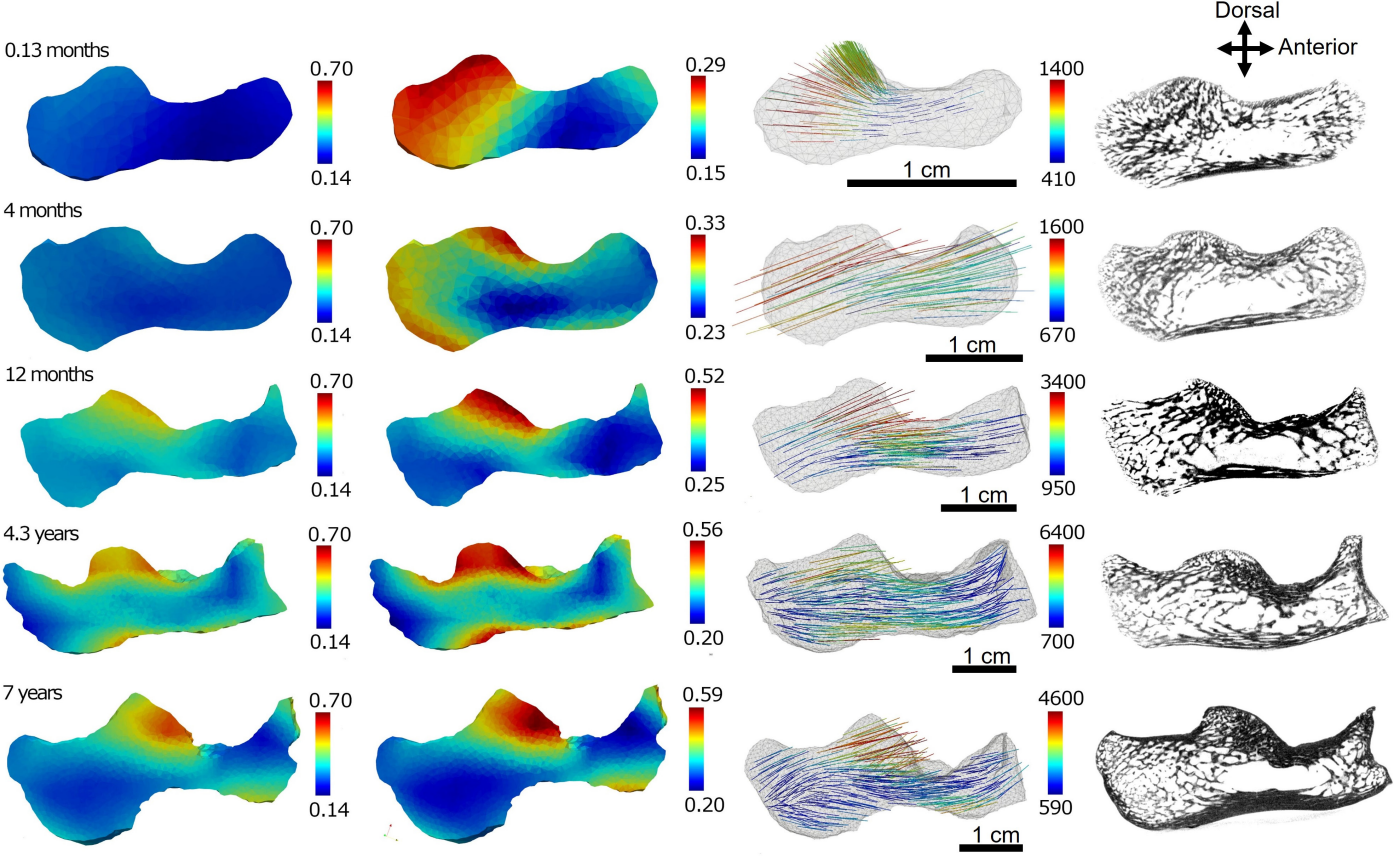
BV/TV scaled between the sample minimum of 0.14 and maximum of 0.70 (left). BV/TV scaled to the minimum and maximum of each individual calcaneus (center left). Vectors show primary direction and magnitude of Young’s modulus (MPa) for each individual (center right). Slice through μCT scan (right). Bones are not to scale; all slices are mid‐sagittal, *n* = 34. *n* = 34. μCT, micro‐computed tomography; BV/TV, bone volume fraction; Tb.Th, trabecular thickness; Tb.Sp, trabecular separation

Figure 7 shows the heterogeneous distribution of BV/TV and primary Young’s modulus throughout sagittal cross sections of a subset of specimens. When BV/TV is scaled between the sample minimum of 0.14 and maximum of 0.70, trabecular structure appears relatively homogeneous at birth and becomes more varied regionally with age. When the colormaps are scaled to the minimum and maximum values for each individual, regional variation is evident at all stages. BV/TV is greatest directly underneath the joint surfaces, particularly of the posterior talar facet. Trabecular structure becomes more heterogeneous with advancing size and age. With a sampling sphere size of 7 mm, and a length of 16 mm in the smallest specimen, some of the homogeneity of the smallest individuals may be due to the lower number of sampling spheres ‐ interpolated onto a larger number of elements.

**FIGURE 8:**
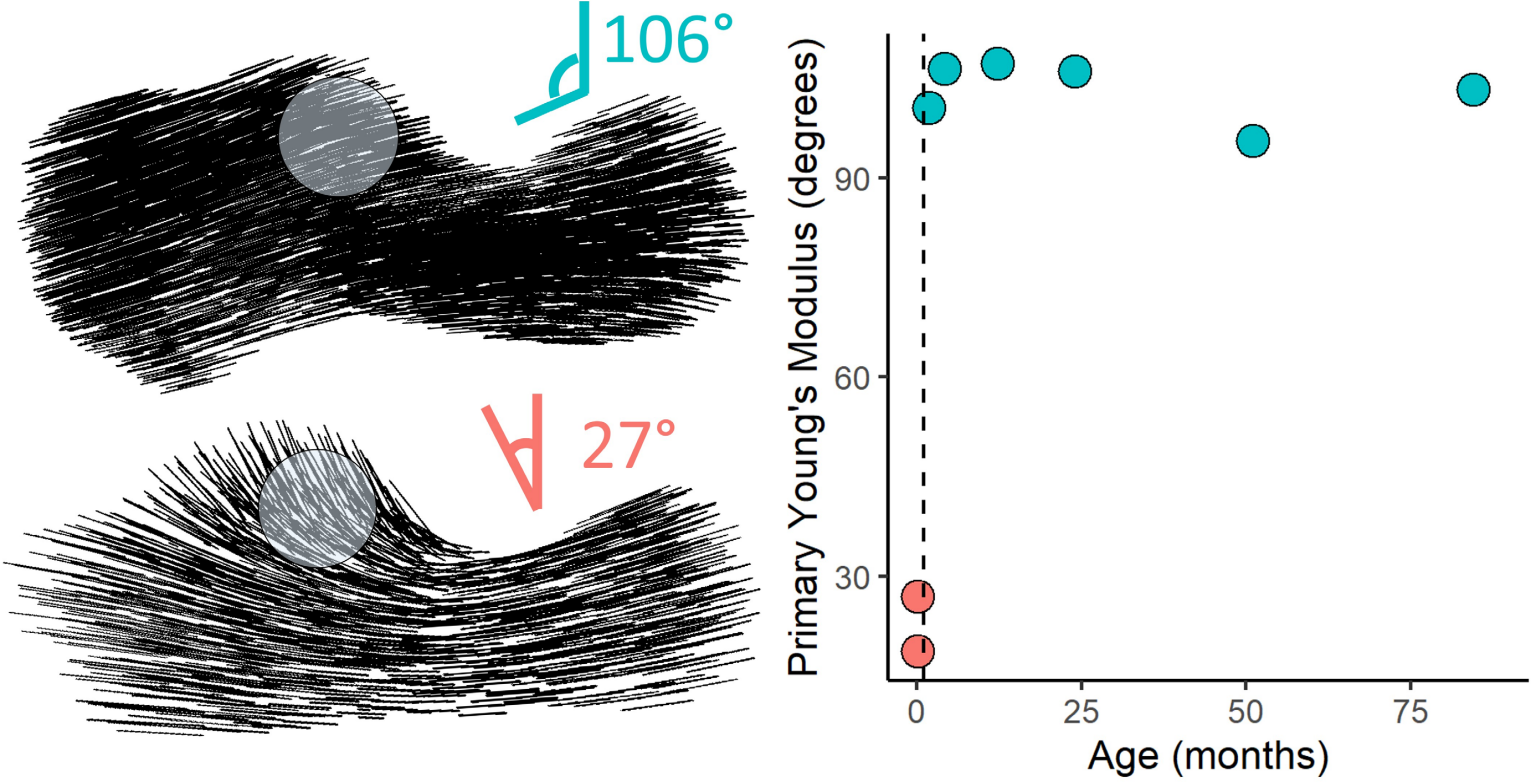
Sagittal slices through the calcaneus with black lines representing the direction in which Young’s modulus is greatest. On the right, the angle of the primary Young’s modulus is plotted against age (months) with the dotted line representing the timing of locomotor onset (~1 month). *n* = 8

The primary Young’s modulus represents the direction of maximum bone stiffness taken at points uniformly distributed throughout the calcaneus (Figures 7 and 8). At birth, primary Young’s modulus is in the direction in which the bone is growing, orthogonal to the edge of the ossification center (Figure 8, bottom left). However, the primary direction of bone stiffness differs in individuals past the first month of age, when the macaques start to locomote regularly (Figure 8, top left). Measured from 12 o’ clock counterclockwise in a sagittal slice through the calcaneus underneath the posterior talar facet, the direction of the primary Young’s modulus changes from 15–35° to 100–110° in all individuals aged greater than 1.8 months of age (Figure 8, right).

### 3.4 Bones and brains

**FIGURE 9:**
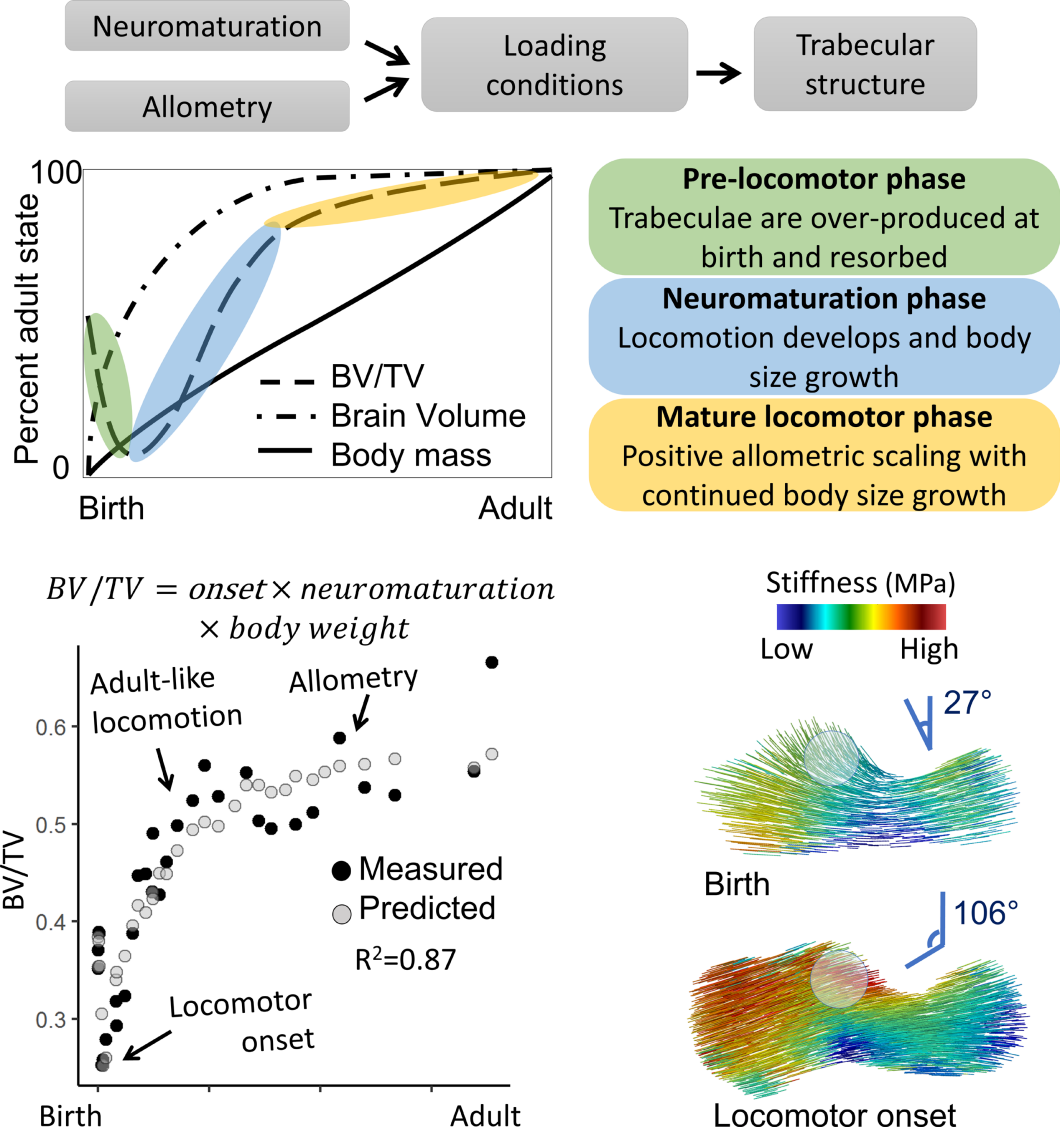
Summary of the paper findings. (Top) Proposed model underlying developmental variation in trabecular structure: Interactions between increasing body mass and increasing neuromuscular control and locomotor experience alter the locomotor loading conditions of the calcaneus, to which the trabecular structure dynamically adapts. (Middle) Three distinct phases of locomotor development can be identified based on changes in the slope of BV/TV with age. (Bottom left) BV/TV plotted against age in months. The black dots represent measured BV/TV, gray dots are predicted BV/TV based on the three‐way interaction between locomotor onset, percent adult brain size, and body mass. This close correspondence supports the model proposed at the top of the figure. (Bottom right) Material stiffness direction and magnitude strikingly differ between the state at birth and after locomotor onset. Presumably, this variation in material stiffness is the result of locomotor loading transmitted through the posterior talar facet of the calcaneus. BV/TV, bone volume fraction

Figure 9 shows the close overlap between attainment of adult brain size and adult‐like BV/TV. While macaques reach their maximum adult body size between 10 and 12 years of age (Hamada, 1994), 95% of maximum brain size is reached at 2 years of age and 100% at 5 years. BV/TV continues to increase slightly after the age when adult brain size has been obtained and body weight continues to increase. We can explain the patterns in Figure 9 with the following model: while the brain is still growing, increases in neuromuscular control and locomotor experience make loading environment of the calcaneus, and by proxy trabecular structure, increasingly like that of adults. When the brain has reached its full size, neuromuscular control of locomotion, and locomotor loading conditions also approach the adult‐like pattern. This model suggests that the steep early age‐related variation in BV/TV found in the macaque calcaneus may be related to increasing neuromuscular control of gait with a slight positive allometry after gait has matured but when body mass continues to increase. We test this model using percent adult brain size as a proxy for neuromuscular control. In Table 4 we compare various types of models to assess which model predicts trabecular properties best (lowest AIC, highest *R* ^2^). Variation in BV/TV is best explained by a three‐way interaction between body mass, percentage of adult brain size, and locomotor onset. Trabecular thickness and separation are also explained by a two‐way interaction between body mass and locomotor onset but differences between interaction models are limited as AIC and *R* ^2^ vary little between them.

## 4 DISCUSSION

We made the following predictions:

1. Onset of locomotion: we predicted that the slope of BV/TV with age in our sample should shift from negative to positive, that trabeculae would become thicker on average, and that Young’s modulus should change direction from orthogonal to the ossification center to joint surfaces. *All these predictions are supported*.
2. Appearance of adult‐like locomotor repertoire: the greatest changes in locomotor anatomy and repertoire occur in the first 12–18 months of life. We predicted that the slope of the relationship between age and BV/TV would become shallower after 18 months of age and largely follow allometric scaling with still increasing body size. *This prediction is supported*.
3. Neuromuscular maturation of gait: We predicted that trabecular properties at different ages should be predictable by an interaction between body mass and percent adult brain size. *This prediction is supported in BV/TV after adding in an extra interaction term for locomotor onset. The prediction is not supported for average trabecular thickness or separation*.

The developmental trajectories of trabecular properties in the calcaneus of Japanese macaques resemble those of other mammals (Colombo et al., 2019; Ragni, 2020; Tsegai et al., 2018; Wolschrijn & Weijs, 2004) including humans (Gosman, 2007; Raichlen et al., 2015; Ryan et al., 2017; Ryan & Krovitz, 2006; Saers et al., 2020), indicating a generally shared mechanism of growth (Carter & Beaupré, 2001). The distribution of BV/TV is substantially more homogenous in younger individuals and becomes increasingly heterogeneous with age, like results reported by Tsegai et al. (2018) for the chimpanzee postcranium. The development of trabecular structure of the calcaneus of Japanese macaques follows the same patterns as the human calcaneus, but with differences in the timing of stages (Saers et al., 2020), as well as other postcranial elements (Acquaah et al., 2015; Gosman & Ketcham, 2009; Raichlen et al., 2015; Ryan & Krovitz, 2006; Saers et al., 2020). In both species, bone is overproduced during early development with high BV/TV and struts largely oriented perpendicular to the ossification center in the calcaneus, or the growth plate in long bones. In both species, BV/TV reduces after birth and begins to increase again at the same time when individuals typically begin independent locomotion. At the same time the primary Young’s modulus shifts in direction from orthogonal to the ossification center to orthogonal to the joint surfaces. This reorientation in the direction in which the bone is loaded helps to more efficiently distribute the loads placed upon the calcaneus during locomotion (Maquer et al., 2015; Roux, 1881; Wolff, 1867; Zysset, 2003). Contrary to our findings, Tsegai et al. (2018) did not find the initial overproduction of trabecular bone, followed by a drop in BV/TV. However, their sample included two individuals between 0 and 5 months of age and it is unclear if they were newborns or already locomoting five‐month‐olds.

Previous work in the human calcaneus found that Tb.Th was largely predictable by increasing body mass throughout ontogeny (Saers et al., 2020), and our results are consistent with this observation. However, we do report for the first time a clear breakpoint in this trend where the slope between body mass and Tb.Th becomes shallower around 17 months of age. This age corresponds to the end of the period of the greatest development in locomotor anatomy and neuromuscular control (the first 18 months of life, Turnquist & Wells, 1993; Wells & Turnquist, 2001), and the eruption of the first permanent molars (18 months, Smith et al., 1994).

The relationship between Tb.Sp and age in the macaque calcaneus resembles that reported for the human calcaneus (Saers et al., 2020), humerus, and femur (Ryan et al., 2017). The average distance between trabeculae increases rapidly after birth (and the start of ossification). After this initial increase Tb.Sp continues to increase slowly with increasing age and body size. However, there is substantial individual variation in Tb.Sp of which roughly half can be explained by allometry (*R* ^2^ = 0.54). The large amount of variation in Tb.Sp relative to Tb.Th and BV/TV areh consistent with results reported throughout the postcranium of humans (Gosman & Ketcham, 2009; Raichlen et al., 2015; Ryan et al., 2017; Saers et al., 2020), apes (Ragni, 2020), and ungulates (Gorissen et al., 2016).

### 4.1 The link between bones and brains

Locomotor patterns are transformed from an immature state to increasingly adult‐like patterns during development. Changes in loading patterns with advancing age are generated by neural circuits that develop in parallel to increases in physical size and weight of a growing organism. Our results lend strong, but indirect support, for a model where trabecular structure is the product of age‐related variation in loading conditions (Figure 9). These changes in loading conditions are a product of the development of gait which, in turn, is the product of neural maturation with age and experience. If this link is demonstrated experimentally, and in other species, trabecular structure could be used as a proxy not only for just development of locomotion but also neural maturation in fossil species. This brain–bone connection would then serve as a powerful life history marker in fossil species.

### 4.2 Developmental trajectories of trabecular structure as a life history marker

We used piecewise regression analysis to locate potential inflection points in the relationship between age and trabecular properties. All identified inflection points occur around two age points: one around 1 month of age (BV/TV, Tb.Sp), and the second around 17 months (BV/TV, Tb.Th). The first inflection point coincides with the period of locomotor onset in Japanese macaques which occurs on average around the end of the first months of life. The second inflection point corresponds to the end of the period of the greatest changes in locomotor anatomy and behavioral control in macaques (18 months of life; Turnquist & Wells, 1993; Wells & Turnquist, 2001) and the eruption of the first permanent molars (18 months, Smith et al., 1994). While some aspects of human bone morphology are genetically determined, others are environmentally induced. For example, the human lateral patellar lip is present already at birth (Lovejoy, 2007; Scheuer & Black, 2004), whereas the human bicondylar angle develops postnatally in response to mechanical loading associated with bipedal locomotion (Tardieu, 1999, 2010; Tardieu & Trinkaus, 1994). In terms of trabecular bone, Barak et al. (2011) showed experimentally that differences in peak loading angle as well as magnitude alter trabecular bone orientation and BV/TV in sheep. Our ontogenetic data also suggest that age‐related variation in trabecular structure corresponds to variation in loading conditions during landmark events in the maturation of gait (onset of locomotion, achievement of adult‐like gait). As such, our results suggest that trabecular structure can potentially be used to infer the timing of locomotor onset and the achievement of adult‐like locomotor repertoires. The advent of independent locomotion coincides with important aspects of mammalian brain size and neuromuscular development (Anderson et al., 2013; Campos et al., 2000; Garwicz et al., 2009). Patterns of locomotor development may therefore provide unique insights into the evolution of locomotor mode, how locomotion develops, neuromaturation, and the onset of independent locomotion in fossil species (Figure 9).

## 5 Limitations

The data presented here correspond to a model where brain maturation increases neuromuscular control of gait, which, in turn, affects the mechanics of gait, which then shape loading patterns of the foot to which trabeculae dynamically adapt. However, our study is cross‐sectional with a skeletal sample of individuals of whom we do not know the individual behavior during life. As such we cannot quantify the changes in mechanical loading over time directly. This study design cannot be used as evidence of a causal mechanism. Confirming our proposed model will require controlled experiments resulting in longitudinal data, ideally in several species.

The percentage of adult brain size is a very rough proxy for neural development and numerous changes in brain composition and wiring occur after adult brain size has been reached (Lebel & Beaulieu, 2011). However adult brain size in humans (calculated from Cofran & Desilva, 2015) is perfectly correlated (*R* ^2^ = 0.99) with experimentally derived measures of neuromaturation (Vaughan & Langerak, 2003). This simple measure of percentage adult brain size is all that is available in paleontological contexts, and it is, therefore, encouraging that we can report such tight correlations.

## 6 CONCLUSIONS

The developmental trajectories of trabecular properties in the calcaneus of Japanese macaques are similar to other species, indicating a broadly shared mechanism of growth. Trabeculae are overproduced at birth, followed by refinement leading to adaptation to local conditions and resulting in a species and joint‐specific heterogeneous state. The end of the first month of life, when the macaques begin to regularly locomote, coincides with striking changes in trabecular structure. This includes a sharp increase in BV/TV and a reorientation of the primary Young’s modulus from orthogonal to the edge of the ossification center to the direction of joint surfaces. The results indicate that age‐related variation in trabecular structure closely corresponds to the onset of independent locomotion and the achievement of an adult‐like locomotor repertoire in Japanese macaques. Locomotor onset coincides with important aspects of mammalian brain size and neuromuscular development and provides a proxy for the degree of precociality of a species. Such bony markers of locomotor development could therefore be compared with other developmental milestones that track the overall pace of life history in fossil species. If the model presented in this paper holds up under longitudinal experimental conditions, trabecular structure can be used both to reconstruct locomotor ontogeny, neuromuscular maturation, and life history in fossil species.

## CONFLICT OF INTEREST

The authors have no conflicts of interest to declare.

## AUTHOR CONTRIBUTIONS

Jaap P. P. Saers: concept/design, funding acquisition, data acquisition and analysis, drafting of the manuscript. Adam D. Gordon: critical revision of the manuscript and statistical advice. Timothy M. Ryan, Jay T. Stock: critical revision of the manuscript.

## Data Availability

The data that support the findings of this study are available on request from the corresponding author.
